# Enhancing cellular behavior in repaired tissue via silk fibroin-integrated triboelectric nanogenerators

**DOI:** 10.1038/s41378-024-00694-5

**Published:** 2024-05-24

**Authors:** Zhelin Li, Shuxing Xu, Zijie Xu, Sheng Shu, Guanlin Liu, Jianda Zhou, Ding Lin, Wei Tang

**Affiliations:** 1 Changsha Aier Eye Hospital, Aier School of Ophthalmology, Central South University, Changsha, Hunan China; 2 The Xiangya Hospital, Central South University, Changsha, Hunan China; 3 Beijing Institute of Nanoenergy and Nanosystems, Chinese Academy of Sciences, Beijing, 101400 China; 4 Center on Nanoenergy Research, School of Physical Science & Technology, Guangxi University, Nanning, 530004 China; 5 School of Nanoscience and Technology, University of Chinese Academy of Sciences, Beijing, 100049 China; 6 Department of Plastic Surgery, the Third Xiangya Hospital, Changsha, Hunan China

**Keywords:** Environmental, health and safety issues, Electrical and electronic engineering

## Abstract

Triboelectric nanogenerators (TENGs) have emerged as a promising approach for generating electricity and providing electrical stimuli in medical electronic devices. Despite their potential benefits, the clinical implementation of TENGs faces challenges such as skin compliance and a lack of comprehensive assessment regarding their biosafety and efficacy. Therefore, further research is imperative to overcome these limitations and unlock the full potential of TENGs in various biomedical applications. In this study, we present a flexible silk fibroin-based triboelectric nanogenerator (SFB-TENG) that features an on-skin substrate and is characterized by excellent skin compliance and air/water permeability. The range of electrical output generated by the SFB-TENG was shown to facilitate the migration and proliferation of Hy926, NIH-3T3 and RSC96 cells. However, apoptosis of fibroblast NIH-3T3 cells was observed when the output voltage increased to more than 20 V at a frequency of 2 Hz. In addition, the moderate electrical stimulation provided by the SFB-TENG promoted the cell proliferation cycle in Hy926 cells. This research highlights the efficacy of a TENG system featuring a flexible and skin-friendly design, as well as its safe operating conditions for use in biomedical applications. These findings position TENGs as highly promising candidates for practical applications in the field of tissue regeneration.

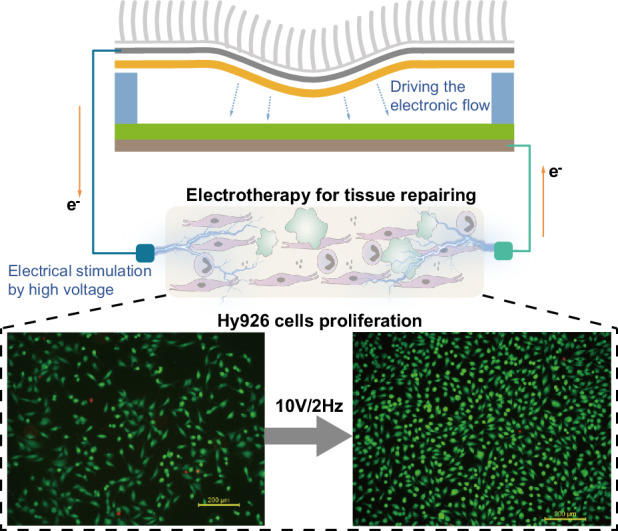

## Introduction

Skin injuries, such as trauma, microbial infections, and thermal damage, have a significant impact on both individual patients and the healthcare system^1–3^. Therefore, these issues have become a global concern in medicine. During the process of skin repair, bioelectrical phenomena occur, which play a crucial role in generating an endogenous bioelectric field that participates in fundamental pathological processes for tissue repair and regeneration^4–7^. The transepithelial electric potential (TEP) generated in this bioelectric field can guide the relevant cells to migrate toward the center of the skin lesion through the ion flow generated by Na^+^/K^+^-ATPase pumps^8^. This process promotes skin repair. Electrical stimulation therapies can enhance the beneficial effect of TEP on wound healing through various strategies^9^. However, the clinical application of electrical stimulation therapy has limitations because it requires patient hospitalization and relies on frequent battery replacement or recharging. Wearable triboelectric nanogenerator (TENG) devices^10–12^, which generate energy through human body motion and friction to output bidirectional electrical signals, offer a more convenient and sustainable power source for facilitating long-term tissue repair^13,14^. In addition, nanogenerators are lightweight and compact, making them suitable for integration into various wearable medical devices without adding significant weight or bulk for a patient to manage.

TENG-generated electrical signals have shown remarkable results in treating wounds, including accelerating wound healing and promoting the proliferation of L929 cells^15,16^. Despite various advances, the skin compliance and comfort of wearable TENGs for wound repair have rarely been systematically researched. Conventional wearable on-skin TENG devices that use synthetic polymer-based substrates such as polyimide^17^, polyvinylidene fluoride^18^, and polyethylene terephthalate^19,20^ have poor air/water permeability, which can cause skin damage^21^. Silk fibroin^22^, a promising flexible substrate material, has emerged in the field of on-skin electronics because of its great mechanical compliance and outstanding properties, such as ideal air/water permeability, excellent skin compliance and controlled biodegradability. Additionally, TENGs are distinguished from conventional external power sources in electrical stimulation systems due to their unique output characteristics, such as high voltage and low current. The generated output varies under different conditions. The wound repair process involves the participation of various cell types that exhibit pleiotropic functions and diverse interactions^16^. To ensure the safe practical application of TENGs, it is crucial to determine the optimal output range and effective stimulation methods^23^. Therefore, further investigation into how cells respond to distinct TENG electrical stimuli is imperative. Corresponding understandings would illuminate the potential impacts of TENG stimulation at each stage of skin wound healing. Furthermore, conventional wearable TENG devices necessitate intentional actions for energy generation. In contrast, the integration of hair-like micropillars^24,25^ allows for the harnessing of energy from subtle mechanical vibrations, such as wind, clothing friction, and natural movements. This unique feature makes TENGs particularly well suited for consistently generating electrical energy during daily activities^26,27^

In this study, we introduce a silk fibroin-based triboelectric nanogenerator (SFB-TENG) characterized by excellent skin compliance and air/water permeability. The device incorporates silk fibroin, polydimethylsiloxane (PDMS), titanium foil and Ag nanowire electrodes. Our study systematically evaluated the impact of electrical stimulation from SFB-TENGs on key biological processes, including proliferation, migration, and cell death, in Hy926, NIH-3T3, and RSC96 cells, which are pivotal to the wound healing process. The results of our research establish a safe and optimal range for generating TENG electrical stimulation outputs that are specifically tailored for vascular endothelial cells, fibroblasts, and nerve cells. This robust experimental foundation supports the potential clinical applications of TENG therapy. Notably, our observations revealed that optimized electrical output conditions effectively promoted the cell cycle progression of vascular endothelial cells, revealing a novel mechanism for TENG electrotherapy. Furthermore, by integrating an Ecoflex micropillar array onto the SFB-TENG, our system can convert diverse human activities into electrical signals of varying magnitudes. This highlights the potential for achieving practical application of SFB-TENGs in various scenarios.

This study addresses the theoretical and practical limitations of wearable on-skin TENG devices in health care and provides new insights into the promising application of TENGs for wound repair and tissue regeneration.

## Results and discussion

### Design of a triboelectric nanogenerator system

**Fig. 1:**
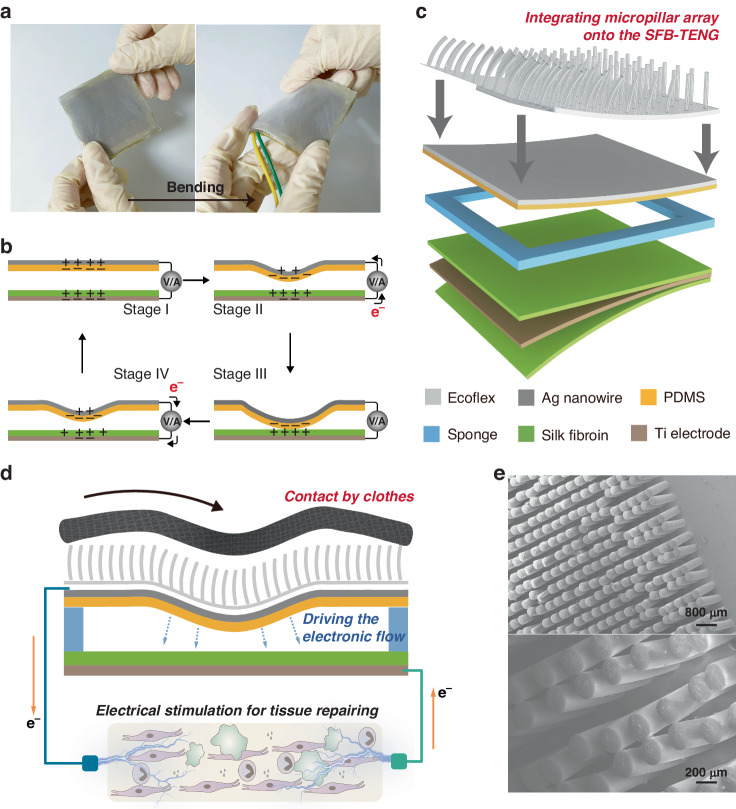
. **Concept, structure and working mechanism of the silk fibroin-based triboelectric system**. **a** Photo of a flexible and biocompatible SFB-TENG. **b** Working mechanism of the four stages of charge transfer in the SFB-TENG. **c** Typical structure of the SFB-TENG. **d** Schematic diagram of the SFB-TENG with a micropillar array. **e** SEM image of the micropillar array. The dimensions are 30 µm in diameter and 180 µm in height

A flexible and biocompatible TENG system was designed to explore the behavior of various types of cells participating in the entire wound repair process, as schematically illustrated in Fig. 1a, c. This system mainly consists of a silk fibroin-based TENG (SFB-TENG). To ensure that the SFB-TENG can conform to the appearance and deformation of skin on all parts of the human body, we utilized thin films of a silicone layer, silk fibroin, and titanium foils to create a fully flexible structure. This fully flexible electrode/friction layer comprised the upper Ag nanowire/PDMS layer based on a manufacturing process as shown in Fig. S1, Supporting Information and Methods section. This layer deforms in the space separated by the 1 mm high sponge under vertical force, generating contact friction with the underlying silk fibroin layer (with a thickness of 50 µm) and thus generating triboelectric charges. The bottom layer of the SFB-TENG consists of a 0.02 mm thick titanium electrode completely covered by two layers of silk fibroin, which constitute the electrode for charge exchange together with the Ag nanowire layer. All materials used to construct the integrated system are easily obtained and biocompatible.

The working mechanism of charge transfer in the SFB-TENG is shown in Fig. 1b. Contact and separation movements between the materials generate electrical charges (contact-electrification), with silk fibroin as the positive tribomaterial losing electrons and PDMS as the negative tribomaterial gaining electrons. In stage I, due to electrostatic induction, corresponding positive charges are induced on the Ag nanowire by the PDMS films, while negative charges on the titanium electrodes are induced by the silk fibroin films. When entering stage II, external forces induce the PDMS layer to move closer to the SF layer, resulting in the generation of current flows from the Ag nanowire layer to the titanium electrodes. Subsequently, the charges and the current on these electrodes gradually decrease to zero after the two layers contact each other in stage III. At the end of the pressing and releasing cycle, the current flows from the titanium electrodes to the Ag nanowire, while the induced charges increase on the electrodes as the PDMS layer moves away from the underlying structure. The multilayer structure then returns to its original state and enters stage I again from stage IV, completing an entire cycle to produce an electrical signal. Its mechanical flexibility is demonstrated by bending the device (Fig. S2). Additionally, we calculated the potential distribution of the SFB-TENG at different phases during friction by the COMSOL 2D model (Fig. S3). The modeling process takes into account the actual size of the device and assigns materials to the model to simulate the potential distribution in the open circuit state.

In addition, to ensure the practical application of the SFB-TENG, we incorporated a micropillar array made of Ecoflex onto the top surface of the SFB-TENG (as depicted in the scanning electron microscope image in Fig. 1e, with the diameter and adjacent distance of the micropillars measuring 30 µm and a height of 180 µm). When the device is applied to the human body, the micropillar array serves as an amplifier for contact stimulation, transferring horizontal interaction forces to the SFB-TENG through clothing rubbing (Fig. 1d). A photograph of the assembled SFB-TENG with micropillars on the forearm of a participant is shown in Fig. S4.

### Fundamental characteristics of the silk fibroin-based triboelectric system

**Fig. 2:**
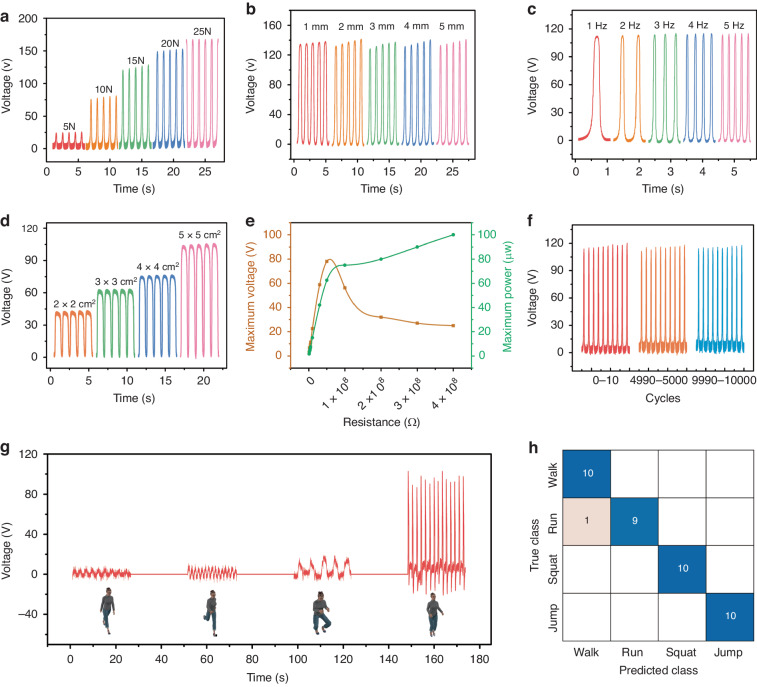
. **Electrical characteristics of the triboelectric generators**. **a** Open-circuit voltage obtained by pressing the 5 × 5 cm^2^ TENG at a frequency of 1 Hz with different forces (from 5 N to 25 N). **b** Open-circuit voltage obtained from TENGs with different separation distances with a force of 15 N at a frequency of 1 Hz. **c** Open-circuit voltage output of the 5 × 5 cm^2^ TENG with a force of 15 N at the pressing frequency (from 1 Hz to 5 Hz). **d** Open-circuit voltage output obtained from TENGs with different areas with a force of 15 N at a frequency of 1 Hz. **e** Maximum voltage curves and the maximum output power for the triboelectric generator with different external load resistances (ranging from 10^6^ to 4 × 10^8^ Ω) and a force of 15 N at a frequency of 2 Hz. **f** Mechanical durability test for up to 10,000 press-release cycles. **g** The signal output of the micropillar-based TENG on the arm when walking, running, squatting and jumping. **h** The confusion matrix for four types of body motion signals

The electrical output performance of the TENG is essential for wound stimulation. To assess the electrical potential output of the PDMS and silk fibroin layers, we established an experimental platform using a linear motor and ergometer (Fig. S5a). The SFB-TENG system was pasted on an ergometer, and a linear motor was utilized to provide periodic pressing by consistent mechanical movements at given parameters. Thus, we investigated the impact of various factors, including force, separation distance, frequency and SFB-TENG size, on the output signals. Voltages and charges were acquired by a 5 × 5 cm^2^ SFB-TENG contact surface with different forces varying from 5 N to 25 N at a frequency of 1 Hz. The generated output voltage increased from 24.2 V to 166.4 V (Fig. 2a), and the charge signal increased from 5.7 nC to 27.8 nC (Fig. S5b) as the pressing force increased from 5 to 25 Hz. When the maximum gap distance between the two parts of the SFB-TENG increased from 1 to 5 mm, voltages and currents were acquired with a force of 15 N at 1 Hz using a 5 × 5 cm^2^ structure. Our results demonstrated that the generated voltage signal barely changed (Fig. 2b), while the currents increased slightly (Fig. S5c) with increasing gap distance. The electrostatic induction becomes weak with only a slight increase in the charge on the SFB-TENG electrodes when the gap distance reaches 5 mm. Moreover, the output voltage of the SFB-TENG remained essentially constant with increasing frequency (Fig. 2c), while the current signal increased from 1.6 μA to 2.7 μA when the frequency varied from 1 to 5 Hz (Fig. S5d). In addition, when we increased the SFB-TENG size from 2 × 2 cm^2^ to 5 × 5 cm^2^, the output voltage signal also varied from 41.2 V to 104.2 V (Fig. 2d). The increased size increases the contact surface area, and more transfer charges are generated between the electrodes, resulting in a higher output.

Additionally, the output voltage and power of the triboelectric system with various external load resistances were calculated (Fig. 2e). The results showed that the maximum output power of the SFB-TENG was 79.677 μW with an external resistance of 58.6 MΩ, indicating that the internal resistance was approximately 60 MΩ. We also analyzed the mechanical durability of the SFB-TENG output by continuously pushing it with a force of 15 N at 2 Hz. As shown in Fig. 2f, after 10,000 cycles of consistent pressing, the voltage signal of the 5 × 5 cm^2^ SFB-TENG experienced a slight attenuation (reduction from approximately 118.7 V to 116.3 V), indicating excellent durability.

Furthermore, we attached the SFB-TENG with a micropillar superstructure to the human arm, belly, and leg and conducted tests to evaluate its electrical output from clothing during activities such as walking, jogging, squatting, and jumping (Fig. 2g and Fig. S6). We quantified the amplitude and period of the output signal produced by the SFB-TENG during each activity as eigenvalues in machine learning for motion recognition, achieving a remarkable 100% recognition accuracy. These findings demonstrate that the SFB-TENG with a micropillar superstructure consistently produces output signals of varying degrees across various motions, indicating its potential for use in healthcare monitoring and electrical stimulation therapy applications.

### TENG electrical stimulation promotes migration and proliferation in vascular endothelial cell lines

**Fig. 3:**
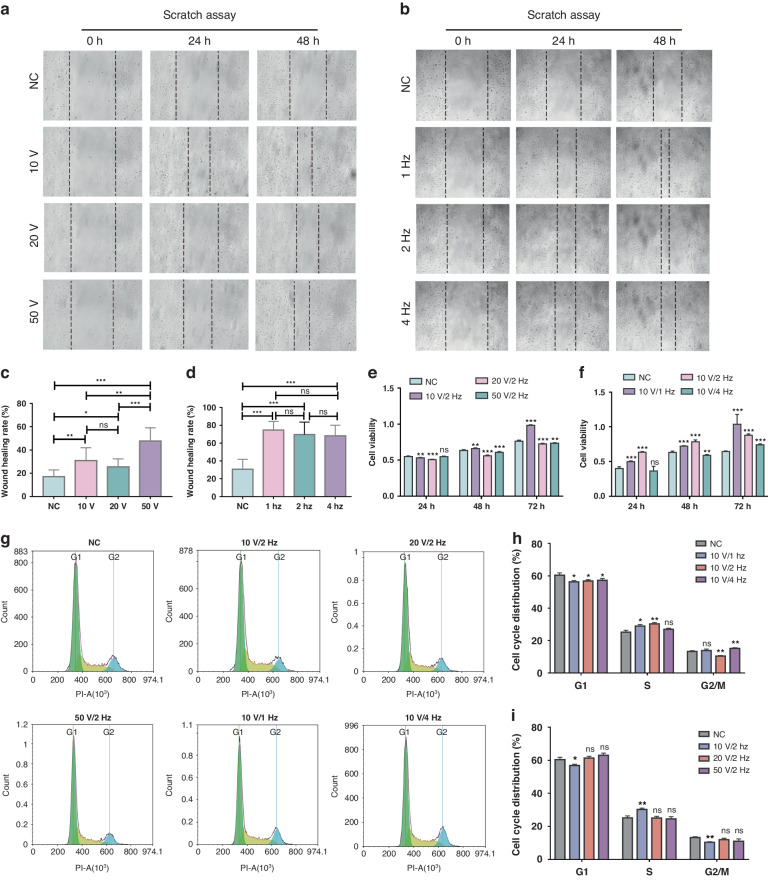
. **Effects of different TENG outputs on the proliferation, migration, and cell cycle progression of hy926 cells**. **a**, **b** The migration properties of the hy926 cells treated with different output voltages (10 V, 20 V, 50 V) and frequencies (1 Hz, 2 Hz, 4 Hz) were determined via a scratch assay. Magnification: ×100. **c**, **d** Quantification of the relative migration rate after various treatments. **e**, **f** CCK-8 results of the hy926 cells at 24 h, 48 h, and 72 h after TENG treatment. **g** Distribution of hy926 cells in different phases of the cell cycle following treatment with different TENG output voltages and different frequencies. **h**, **i** Quantification of the cell cycle distribution after various treatments

During the inflammation and angiogenesis stages of wound healing, vascular endothelial cells play a crucial role in the repair process by proliferating, migrating, and branching to form new blood vessels^16,23^. As illustrated in Fig. 3, the biological behaviors of the vascular endothelial cell line hy926 subjected to electrical stimulation with different output voltages (10 V, 20 V, 50 V) and different frequencies (1 Hz, 2 Hz, 4 Hz) of SFB-TENGs were detected by scratch assays and CCK8 assays. Twenty-four hours after the hy926 cells were seeded into the six-well plates, Au electrodes connected to the SFB-TENG were placed at the end of each well to produce 10 V, 20 V, and 50 V output voltages within the dish or at frequencies of 1 Hz, 2 Hz, and 4 Hz with an output voltage of 10 V after rectification (1 h per day). Images of the migration of the hy926 cells in each group were then acquired after 24 h and 48 h under the impact of different electric fields (Fig. 3a,b). Our results indicate that, in comparison with the negative control group, the hy926 cell experimental groups exposed to varying degrees of electrical stimulation exhibited noticeably faster wound closure (*n* = 9). Furthermore, as the output voltage increased, the group exposed to 50 V and 2 Hz exhibited a significantly greater wound healing rate than did the groups exposed to 20 V at 2 Hz and 10 V at 2 Hz (Fig. 3c). Additionally, the wound healing rates of the hy926 cells at frequencies of 1 Hz, 2 Hz, and 4 Hz were significantly greater than those of the negative control group. However, there were no statistically significant differences observed between the experimental groups at these three frequencies (Fig. 3d). These findings indicate that SFB-TENG significantly enhanced the migration ability of hy926 cells, leading to a wound healing rate that was more than two times greater than that of the control group. However, it was observed that increasing the frequency from 1 Hz to 4 Hz did not yield any statistically significant response.

In addition, we evaluated the proliferation of the hy926 cells by assessing cell viability in the control and experimental groups using a CCK-8 assay after 3 days of electrical stimulation by the TENG (1 h/day). As depicted in Fig. 3e, our findings indicate that the cells in the 10 V/2 Hz group had greater viability (48 h: 0.66 ± 0.004/72 h: 0.9847 ± 0.003) than did those in the negative control group (48 h: 0.6348 ± 0.005/72 h: 0.7632 ± 0.006) after 48 h and 72 h of culture. However, increasing the voltage output while maintaining the frequency slightly inhibited the proliferation of the hy926 cells. We further investigated the potential effect of electrical stimulation by TENGs at different frequencies with a constant output voltage of 10 V. Interestingly, compared to control cells, hy926 cells treated with SFB-TENGs at frequencies of 1 Hz and 2 Hz exhibited greater proliferation rates at 24 h, 48 h, and 72 h, respectively. Additionally, the proliferation of cells in the 4 Hz group significantly increased after 72 h of culture. Among these groups, the hy926 cells treated at a frequency of 1 Hz displayed the highest cell viability (1.036 ± 0.06) after 72 h, while the cell viability in the relevant control groups remained at 0.6447 ± 0.003 (Fig. 3f). These findings indicate that relatively low voltage and frequency are required to promote the proliferation of hy926 cells.

To elucidate the mechanism by which SFB-TENG electrical stimulation promotes cell proliferation, we analyzed the cell cycle distribution of the hy926 cells after 2 days of treatment (Fig. 3g). Our findings demonstrated that treatment with 10 V at frequencies of 1 Hz and 2 Hz resulted in a decrease in the number of hy926 cells in the G0/G1 phase and an increase in the proportion of cells in the S phase. Additionally, consistent with previous results from the cell viability test, there was no significant difference in the distribution of cells in the G1, S, and G2/M phases of the cell cycle when the voltage output of the SFB-TENG treatment was increased to 20 V and 50 V (Fig. 3h, i). These results demonstrate that a relatively low output voltage and frequency can facilitate the cell cycle to stimulate faster proliferation of human vascular endothelial cells in vitro. To assess whether different SFB-TENG treatments could cause damage and induce apoptosis in hy926 cells, we performed calcein-acetoxymethylester (calcein-AM)/propidium iodide (PI) and Annexin V with fluorescein isothiocyanate (FITC)/PI double staining. Calcein-AM/PI staining suggested that electrical stimulation with SFB-TENGs at different voltages and frequencies did not cause additional cell death in the hy926 cells (Fig. S7). This finding was further supported by Annexin V-FITC/PI staining via flow cytometry analysis (Fig. S8), revealing a safe output range for vascular endothelial cells.

### Effects of electrical stimulation by TENGs on the migration and proliferation of NIH-3T3 and RSC96 cells

After early neovascularization is established, contractile resident fibroblasts migrate and proliferate to replace the fibronectin provisional matrix, invade the clot, and initiate collagen synthesis to form granulation tissue at wound sites^28,29^. Mature fibroblasts contribute to increased collagen deposition as well as the initiation of wound contraction by differentiating into myofibroblasts^30,31^. Nerve regeneration and collateral reinnervation are two primary processes involved in the restoration of peripheral nerves. These processes are completed through the regrowth of two myelinated nerve fiber stumps and the reconnection of damaged nerve tissue terminals^16^. In the remodeling stage, Schwann cells play an essential role in these processes by interacting with ephrin-B in fibroblasts and migrating to the ends of nerve stumps^32^, guiding axon growth through newly aligned vasculature. To explore the impact of SFB-TENG electrical stimulation at different output voltages and frequencies on fibroblasts and Schwann cells, we next assessed the cellular responses of the NIH-3T3 and RSC96 cell lines.

**Fig. 4:**
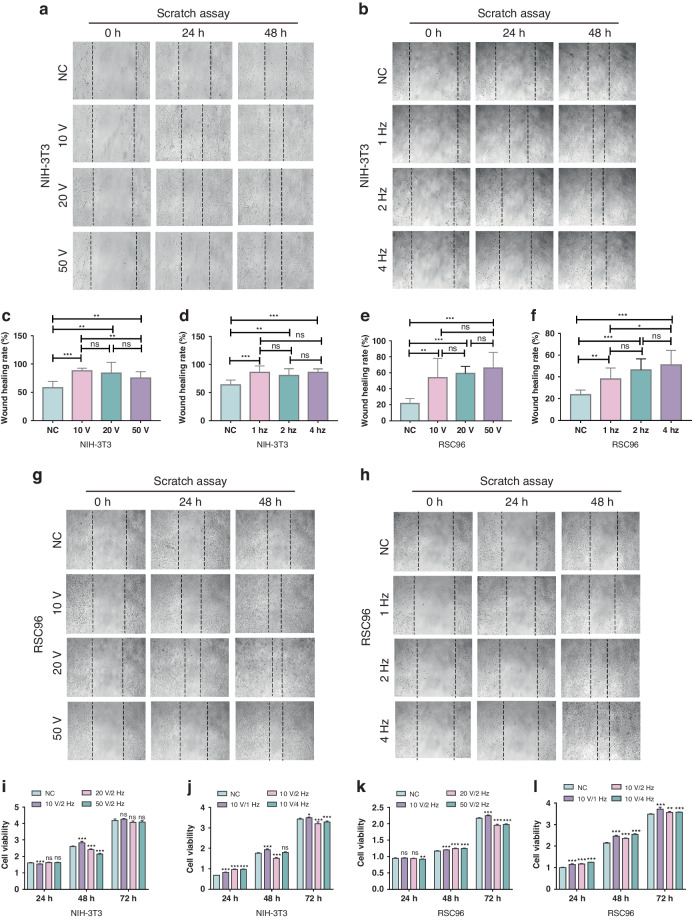
. **Effect of different TENG outputs on the proliferation and migration of NIH-3T3 and RSC96 cells**. **a**, **b** The migration properties of NIH-3T3 cells treated with different output voltages and frequencies were determined via a scratch assay. Magnification: ×100. **c**, **d** Quantification of the relative migration rate of NIH-3T3 cells after TENG treatment. **e**, **f** Quantification of the relative migration rate of RSC96 cells after TENG treatment. **g**, **h** The migration properties of NIH-3T3 cells treated with different output voltages and frequencies were determined via a scratch assay. Magnification: ×100. **i**, **j** CCK-8 results for NIH-3T3 cells at 24 h, 48 h, and 72 h after TENG treatment. **k**, **l** CCK-8 results for RSC96 cells at 24 h, 48 h, and 72 h after TENG treatment

Our scratch assay results indicated that SFB-TENG stimulation significantly promoted the migration rate of both NIH-3T3 cells (Fig. 4a, b) and RSC96 cells (Fig. 4g, h) compared to that in the negative control group at 48 h. Quantitative analysis of NIH-3T3 cells demonstrated that when the output voltage reached 50 V at a frequency of 2 Hz, the migration-promoting effect was slightly decreased (75.39 ± 3.753%) compared to that in the 10 V/2 Hz group (88.19 ± 1.453%) (Fig. 4c). However, for NIH-3T3 cells treated with 10 V at three different frequencies, there was no significant difference in the migration-enhancing effect between the groups (Fig. 4d). In contrast, for RSC96 cells, the quantitative results indicated no significant difference between the migration rates of cells in the 10 V, 20 V, and 50 V groups at an output frequency of 2 Hz (Fig. 4e). Moreover, with increasing output frequency, the migration of RSC96 cells markedly accelerated after 2 days of treatment. Specifically, the migration rate of the RSC96 cells treated with 10 V/4 Hz (50.99 ± 4.459%) was significantly greater than that of the cells in the 10 V/1 Hz group (38.06 ± 3.372%) (Fig. 4f). These results indicate that the migration ability of NIH-3T3 fibroblasts tends to be mildly restricted when the output voltage increases, while the migration ability of Schwann cells (RSC96) can be elevated by increasing the output frequency.

In addition, we assessed the proliferation of NIH-3T3 and RSC96 cells after 3 days of SFB-TENG-generated electrical stimulation via a CCK8 assay. In the initial analysis, it was observed that the 10 V/2 Hz, 20 V/2 Hz and 50 V/2 Hz treatments did not consistently affect the proliferation of NIH-3T3 cells (Fig. 4i). After assessing the cell viability at different frequencies with a minimum output voltage of 10 V, our findings showed that when the stimulation frequency decreased to 1 Hz, the cell viability of NIH-3T3 cells slightly increased at 24 h, 48 h, and 72 h compared to that of the control group. Interestingly, after increasing the stimulation frequency again, the proliferation of the NIH-3T3 cells in the 2 Hz group and 4 Hz group was mildly suppressed (Fig. 4j). Therefore, we further analyzed the cell death rate of NIN-3T3 cells to assess its response to SFB-TENG stimulation. The results of calcein-AM double staining revealed that the cell death rate significantly increased after the output voltage reached 20 V and 50 V, while it remained relatively stable when the frequency increased (Fig. S9). These results indicate that mild electrical stimulation with a relatively lower output (approximately 10 V at a frequency of 1 Hz) is optimal for fibroblast-derived NIN-3T3 cells.

For the RSC96 cells, the cell viability under 10 V/2 Hz stimulation increased after 72 h, while the proliferation rate of the other two groups (20 V/2 Hz and 50 V/2 Hz treatment) was inhibited compared to that of the control cells (Fig. 4k). However, the number of RSC96 cells treated with 10 V at different frequencies significantly increased (Fig. 4l). The cells in the 10 V/1 Hz group exhibited the highest cell viability of 3.716 ± 0.04, while the 10 V/2 Hz and 10 V/4 Hz groups showed slightly lower values of 3.561 ± 0.02 and 3.573 ± 0.005, respectively. These results indicate that the most suitable electrical stimulation for facilitating proliferation in RSC96 cells is approximately 10 V/1 Hz. We also determined the apoptosis rate of RSC96 cells in each group. CSFB-TENG did not increase cell death in cells subjected to calcein-AM/PI double staining after electrical stimulation at different output voltages or frequencies, indicating that the electrical stimulus range for Schwann RSC96 cells was safe (Fig. S10).

## Conclusions

In this study, we successfully developed a silk fibroin-based electrical stimulation system designed for therapeutic electrical stimulation in tissue regeneration. The SFB-TENG device demonstrates remarkable biocompatibility with its constituent materials, establishing a resilient connection between its flexible structure and the skin surface. Using the SFB-TENG, we applied electrical stimulation at various frequencies and voltages to cells integral to tissue repair and wound healing processes, including vascular endothelial cells, fibroblasts, and Schwann cells. We further assessed the safety and effectiveness of TENG electrotherapy from an in vivo perspective. Our results indicate that a relatively low output voltage and frequency are suitable for electrical stimuli at the cellular level. Although the rates of vascular endothelial cell and Schwann cell death were negligible after treatment with various SFB-TENGs, fibroblast apoptosis markedly increased as the output voltage increased. Notably, mild electrical stimulation of SFB-TENGs facilitated the cell cycle in vascular endothelial cells. These findings have broader applicability to TENG-based applications and future studies focused on tissue regeneration. In addition, by incorporating micropillars on the surface of the SFB-TENG, this device has the potential to facilitate extended health care monitoring and clinical electrotherapy. This work demonstrates a promising biocompatible electrical stimulation system and establishes a robust experimental foundation for TENG electrotherapy, paving the way for prospective practical applications in the field of tissue repair and regeneration.

## Experimental section

### Fabrication of the SFB-TENG device

#### Synthesis of Ag nanowire films

After mixing the main agent and the curing agent of PDMS (Dowcorning SYLGARD 184) according to the ratio, the solution was vacuumized, spin-coated, and dried to obtain a 100 µm thick PDMS film. The obtained PDMS film was plasma-cleaned (SENTECH SI 500) at a power of 50 W for 1 min in an oxygen-filled environment. Then, the silver nanowire solution was evenly spread on the cleaned PDMS, oxidized, and dried at 100 °C to obtain the Ag nanowire film (Fig. S1).

#### Assembly of the SFB-TENG

Two pieces of silk fibroin were cut and stuck on both sides of the Ti electrode (50 × 50 × 0.02 mm), which were used as the friction material and skin contact layer, respectively. A sponge spacer (with an outer diameter of 5 × 5 cm and an inner diameter of 4 × 4 cm) was made, and silver nanowire films attached to the abovementioned aggregates and Ti were pasted on the two sides to construct a flexible triboelectric nanogenerator prototype. To complete the assembly, wires were drawn from the silver electrode and titanium electrode, which were then connected to the Au electrode for electrical stimulation. This process ensures the integration of all components, culminating in the construction of a functional and flexible triboelectric nanogenerator ready for experimentation.

#### Micropillar manufacturing

Deformation simulation was conducted using COMSOL software with different micropillar sizes. (Fig. S11). The mold was fabricated using computer numerical control (CNC) technology based on a drawing created in Adobe Illustrator 2020. The Smooth-On (Ecoflex0030) components were mixed and stirred for 20 min before being poured into the mold (with a diameter and adjacent distance of 30 µm and a height of 180 µm). After the mixture was vacuumed in the mold, it was dried at 80 °C to obtain the micropillar structure upon demolding. Subsequently, the micropillar array made of Ecoflex was assembled on the top surface of the SFB-TENG and closely adhered to the underlying layer of Ag nanowires.

### Performance of SFB-TENGs

The features of the silk fibroin-based triboelectric nanogenerator were evaluated under various conditions using linear motors from Beijing Nanon Instrument Technology Co., Ltd. These conditions encompassed different pressures, frequencies, intervals, sizes, and other relevant parameters. Subsequently, the output voltage, current, and charge of the SFB-TENG were measured using a 6514 electrometer. For practical examination of the effects of the SFB-TENG on the human body, enhanced by the incorporation of the micropillar superstructure, the SFB-TENG was positioned on the arm, abdomen, and calf to record the electrical signals generated during routine daily activities.

### Analysis of cell migration and proliferation

The Hy926, NIH-3T3 and RSC96 cell lines were obtained from Procell Life Science (Wuhan, China). For the scratch assay, different groups of cells were seeded in 6-well plates (7 × 10^5^ cells/well) and cultured overnight. After the confluence reached approximately 80%, one line was drawn in the middle of each well by using a 1000 µL pipette tip, and the cells were washed with DMEM. After sterilization of the Au electrodes, the gold electrode connected to the negative pole was placed in the middle of the wells, and the gold electrodes connected to the positive pole were placed on both sides of the wells. Then, the electrical stimulation of SFB-TENGs with different outputs was applied after rectification for 1 h/day in each treatment group. Samples without electrical stimulation were considered the control group. Cell migration images were obtained using a Zeiss optical microscope at 100× magnification after 24 and 48 h of culture. The photographs were captured from a fixed position. Subsequently, ImageJ software was used for image processing and to quantify the migration area. The cell migration rate was determined using the 0-h and 48-h datasets with the following formula:

$${\rm{Cell\; migration\; rate}}( \% )=(1-{\rm{scratc}}{\rm{h}}{\rm{area}}/{\rm{original\; scratc}}{\rm{h}}{\rm{area}})\times 100 \% .$$

In the cell proliferation experiments, these cells were seeded at a density of 2 × 10^5^ cells/well on 6-well plates. Then, the electrical stimulation of SFB-TENGs with different outputs was applied after rectification for 1 h/day in each treatment group. Then, 100 μL of CCK8 solution was added to each well after 24, 48, and 72 h. Then, the 6-well plate was kept in an air atmosphere containing 5% CO_2_ at 37 °C for 2 h. An enzyme labeling instrument (Thermo Fisher Scientific, USA) was used to evaluate the OD value of each well at a wavelength of 450 nm to determine the proliferation rate of each group.

### Analysis of the cell cycle and apoptosis

For cell cycle analysis, the cells were collected 48 h after electrical therapy. After washing with phosphate-buffered saline solution, 1 mL of precooled 70% ethanol was added, and the mixture was incubated overnight at 4 °C. Then, the cells were collected again by low-speed centrifugation and suspended in 500 μL of flow cytometry (FACS) buffer supplemented with 2.5 μL of 1.0 mg/mL RNase at room temperature for 15 min. Then, the cells were incubated with 25 μL of propidium iodide (1 mg/mL) at room temperature in the dark for 15 min. The distribution of the cell cycle was detected by flow cytometry analysis (Becton Dickinson, Franklin Lakes, NJ, USA).

In the apoptosis assay, the cells were placed into 6-well plates at a density of 5 × 10^5^/well and grown overnight at 37 °C with 5% CO_2_. The electrical stimulation of SFB-TENGs with different outputs was applied after rectification for 1 h/day in each treatment group. After 48 h, the cells were collected for apoptosis assessment with Annexin-V (FITC) and PI kits (KeyGEN BioTECH, China) and subsequently analyzed by flow cytometry (Becton Dickinson, Franklin Lakes, NJ, USA). Furthermore, the treated cells were stained with calcein and PI (KeyGEN BioTECH, China) after 48 h. Then, a fluorescence microscope was used to obtain optical images.

### Data analysis and statistics

To assess the SFB-TENG output performance, a software platform based on LabVIEW was built for real-time acquisition. Origin 2015 software was used for further processing and analysis of the experimental data. In motion pattern recognition, the subjects who wear the device perform walking, running, squatting, or jumping ten times. The amplitude and period in each motion cycle are extracted as two-dimensional eigenvalues in the calculation process of the support vector machine. Tenfold cross-validation was used to test the training accuracy and stability of the model; that is, nine sets were used for training, and one set was used for verification. The distribution scatter plot, confusion matrix, and ROC curve after training and verification are shown in Fig. S5. The programming software for machine learning was MATLAB 2020.

All biological assays were repeated at least three times. The experimental data are presented as the mean ± standard deviation and were statistically analyzed by GraphPad Prism 7. The quantitative data were compared between different groups by *t* tests or one-way analysis of variance (ANOVA). The results were considered statistically significant if the *P* value was <0.05.

### Supplementary information

Revised Supporting Information with marks

## Data Availability

The original contributions presented in the study are included in the article. Further inquiries can be directed to the corresponding authors.
