# Supplementary material for: Enhancing cellular behavior in repaired tissue via silk fibroin-integrated triboelectric nanogenerators
Source: Microsyst Nanoeng. 2024 May 24;10:68. doi: 10.1038/s41378-024-00694-5 (PMC11126623; doi:10.1038/s41378-024-00694-5)
Supplement: Supplementary file 1 — Revised Supporting Information with marks [file 41378_2024_694_MOESM1_ESM.docx]

Supplementary Material

**Enhancing Cellular Behavior in Tissue Repair based on Silk Fibroin-Integrated Triboelectric Nanogenerators**

*Zhelin Li^1,2^, Shuxing Xu*^3,4,5^*, Zijie Xu^3,5^, Sheng Shu^3,5^, Guanlin Liu^4^, Jianda Zhou^6*^, Lin Ding^1^* and Wei Tang^3,5*^*

Correspondence: Jianda Zhou ([zhoujianda @ csu.edu.cn](mailto:guanlinliu@gxu.edu.cn%20(Guanlin)) or Lin Ding ([linding@csu.edu.cn](mailto:linding@csu.edu.cn)) or Wei Tang ([tangwei@binn.cas.cn](mailto:tangwei@binn.cas.cn))

^1^Changsha Aier Eye Hospital, Aier School of Ophthalmology, Central South University, Changsha, Hunan, China.

^2^ The Xiangya Hospital, Central South University, Changsha, Hunan, China.

^3^Beijing Institute of Nanoenergy and Nanosystems, Chinese Academy of Sciences, Beijing, 101400 P. R. China.

^4^Center on Nanoenergy Research, School of Physical Science & Technology, Guangxi University, Nanning, 530004 P. R. China.

^5^School of Nanoscience and Technology, University of Chinese Academy of Sciences, Beijing, 100049, China.

^6^Department of Plastic Surgery, the Third Xiangya Hospital, Changsha, Hunan, China.


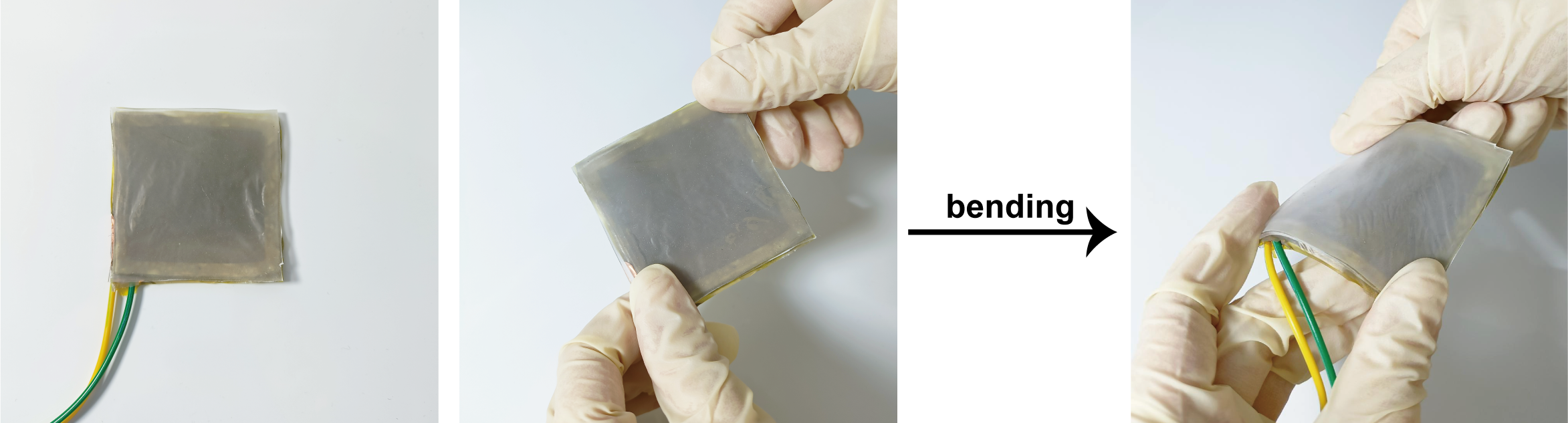


**Figure S1** The photo of a flexible and biocompatible micropillar-based triboelectric nanogenerator (MB-TENG).


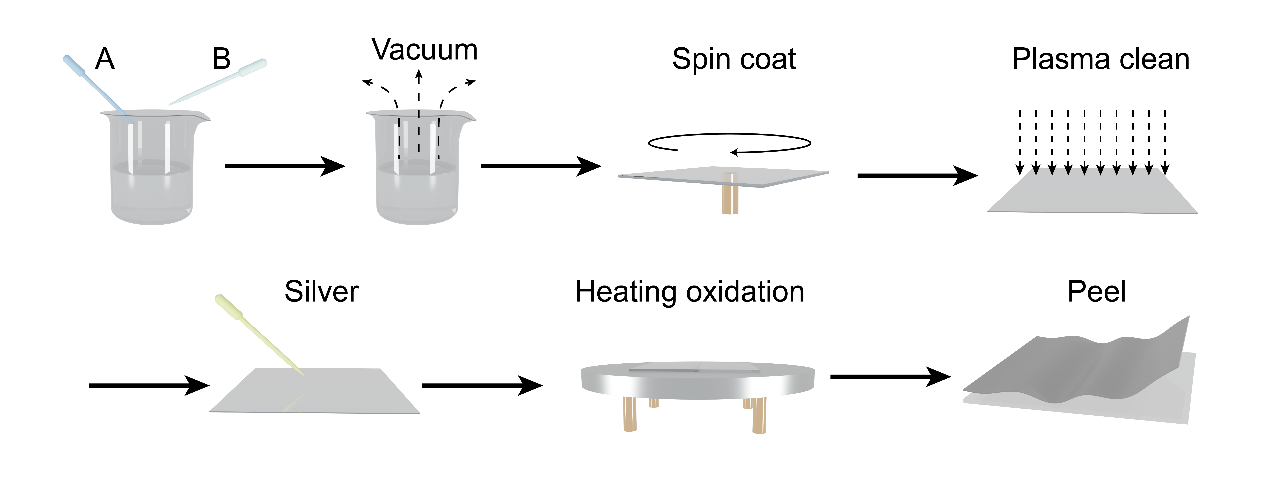


**Figure S1** Flow chart of PDMS-Ag nanowire film fabrication.


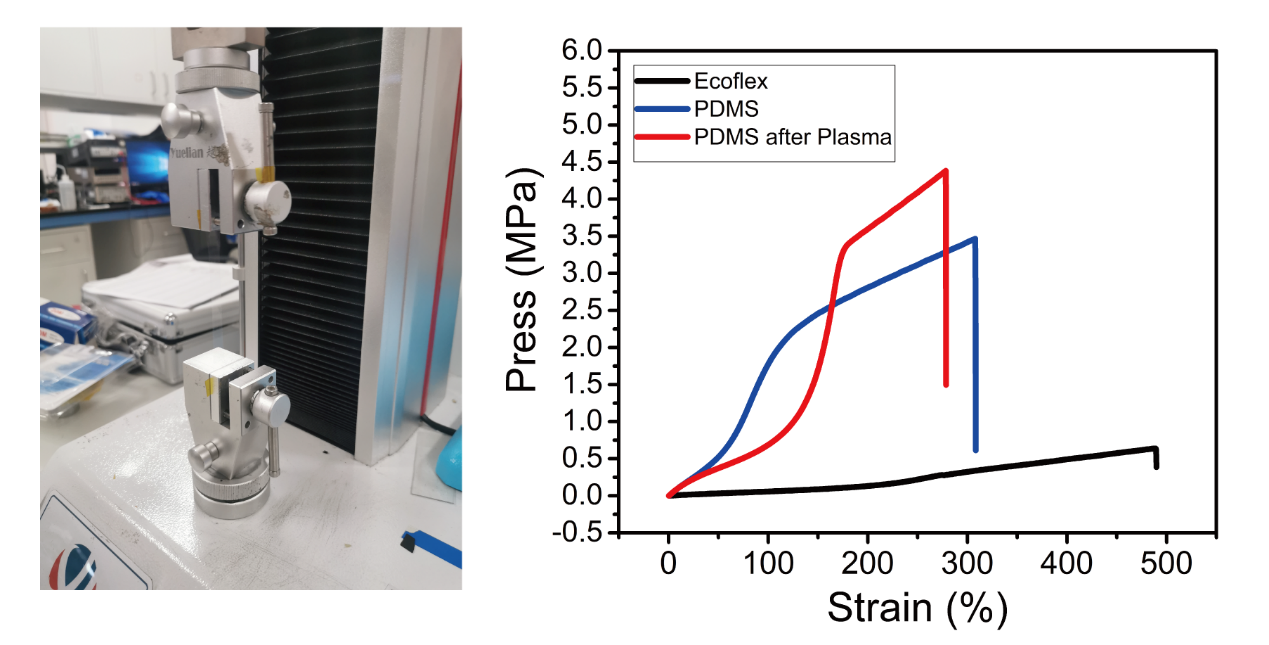


**Figure S2** The stress-strain curves of Ecoflex and treated PDMS film.


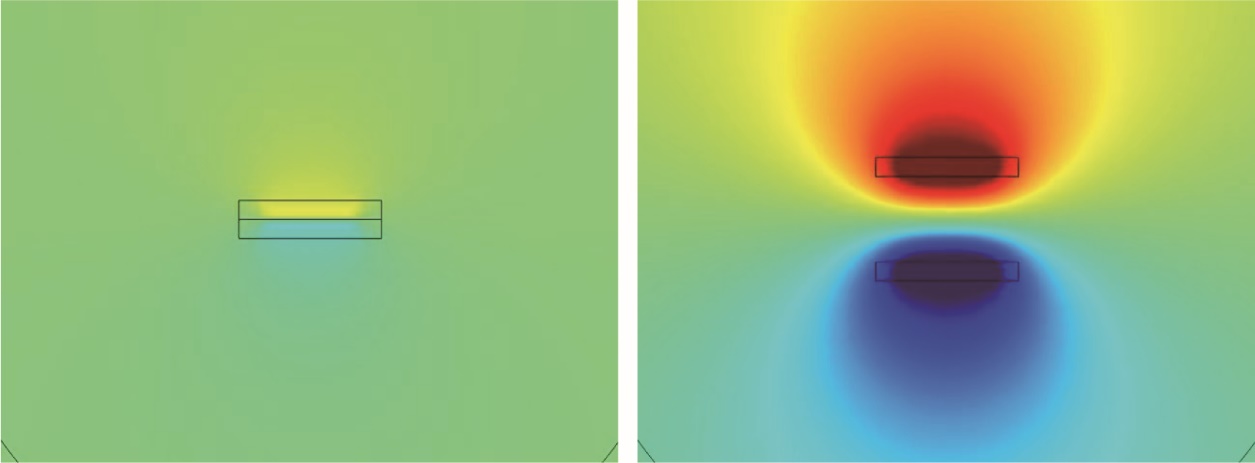


**Figure S3** Finite element simulation using COMSOL to analyze the potential distribution during friction of micropillar-based TENG. In the simulation process, rectangular blocks are first designed based on the prototype dimensions of the device, and then specific materials are assigned to corresponding rectangular blocks. The entire working process is simulated according to the distance between two friction materials during actual operation. From the simulation results, it can be seen that when the two friction materials are in complete contact, there is no potential difference between them because the charges on the surfaces of the two friction materials neutralize each other. However, when they begin to separate, a potential difference appears, and when they are separated to the maximum distance, the potential difference reaches its maximum value.


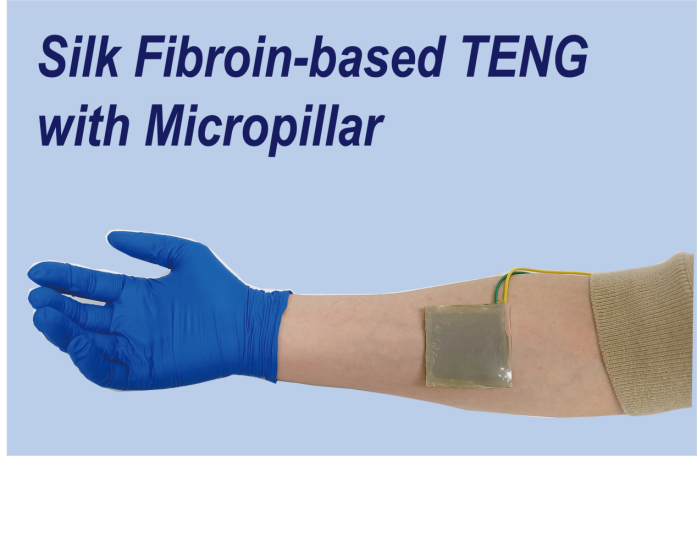


**Figure S4** The photo of a flexible and biocompatible SFB-triboelectric nanogenerator.


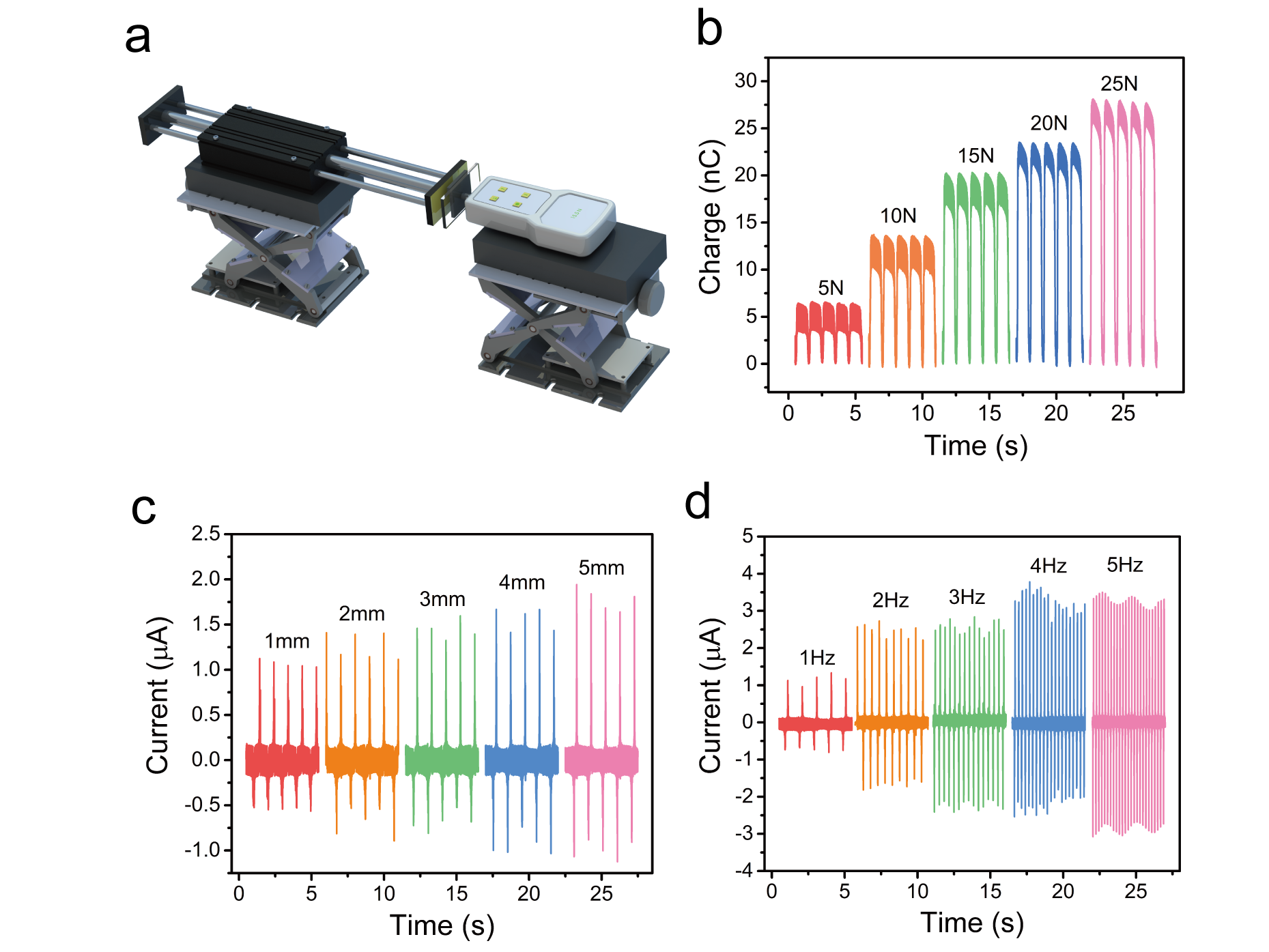


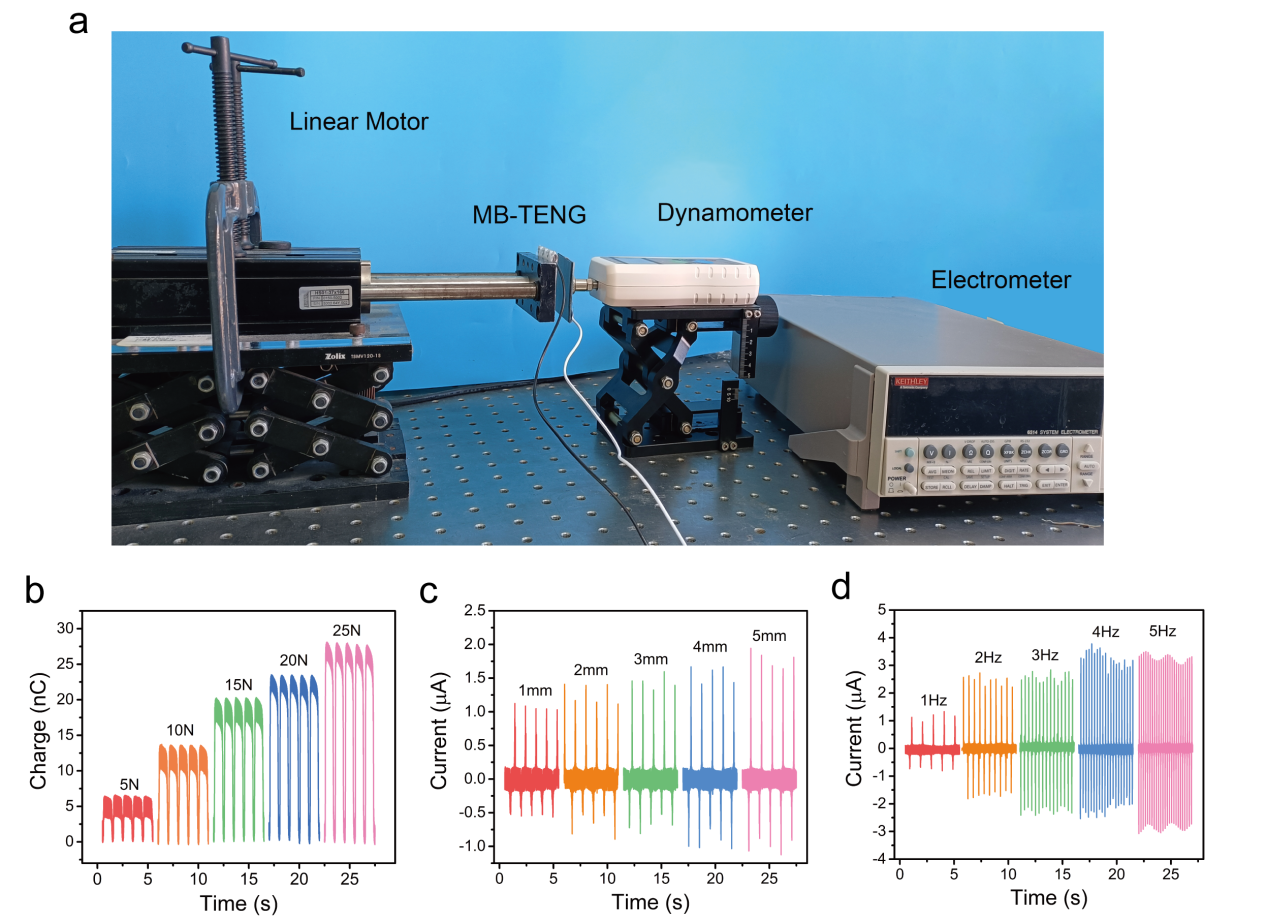


**Figure S5**. Electrical characteristics of the triboelectric generators. **a** The experimental platform containing linear motor and ergometer. **b** The charge obtained by pressing the 5 × 5cm^2^ TENG at 1 Hz frequency with different forces (from 5 N to 25 N). **c** The short-circuit current output obtained from TENG with different Separation distance (1 mm to 5 mm) with a force of 15 N at frequency of 1 Hz. **d** The short-circuit current output of the 5 × 5cm^2^ TENG with a force of 15 N at the pressing frequency (from 1 Hz to 5 Hz).


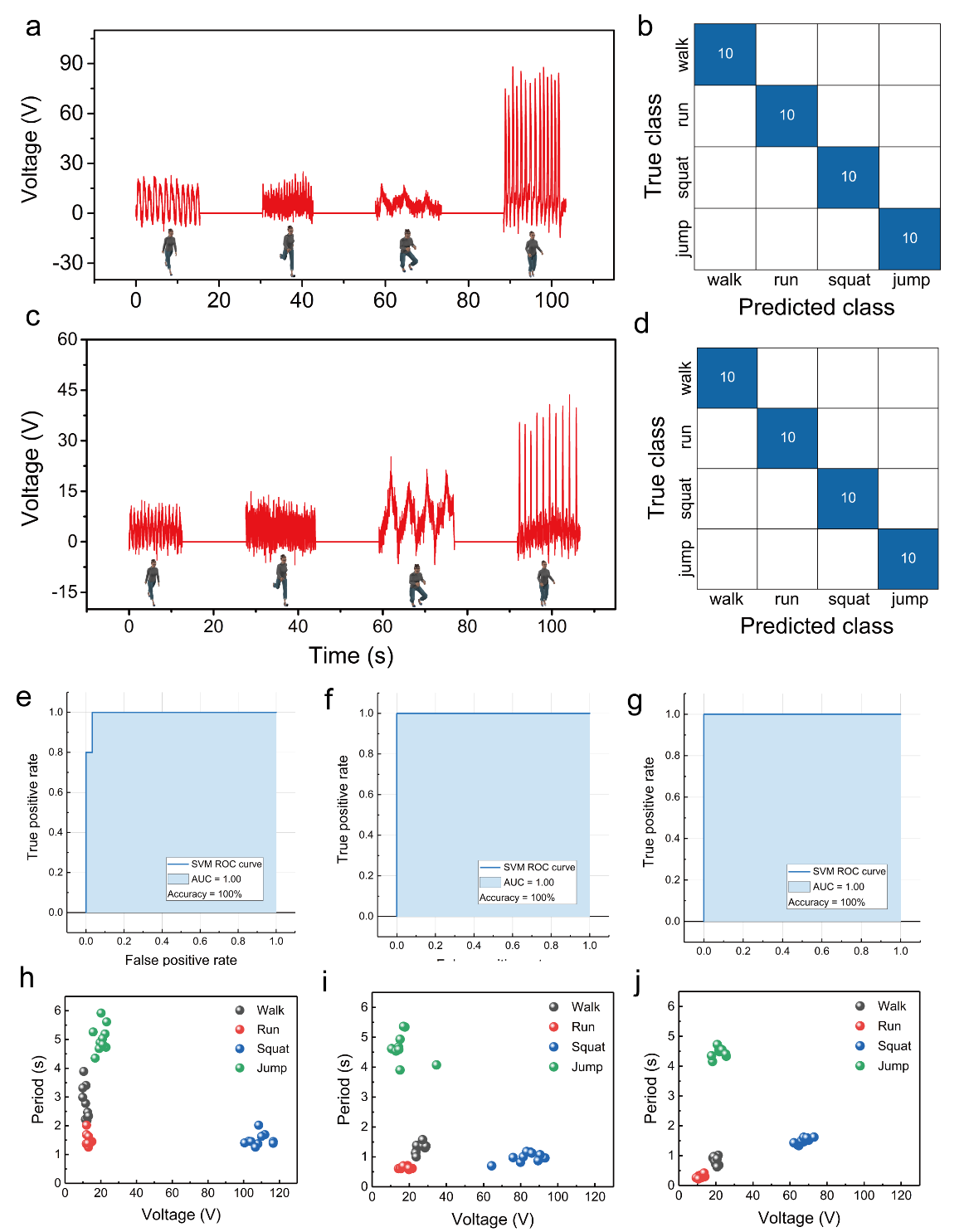


**Figure S6** Signals generated by different movement and a comparison of movement and body-motion signals. **a** The signals output of micropillar-based TENG on the abdomen while walking, running, squatting and jumping. **b** The confusion matrix for four types of body-motion signals when TENG on the abdomen. **c** The signals output of micropillar-based TENG on the lower limbs while walking, running, squatting and jumping. **d** The confusion matrix for four types of body-motion signals when TENG on the lower limbs. **e**, **f**, **g** The SVM ROC curve of distinguishing the different signal from different body-motion while the TENG is on the upper limbs, abdomen and lower limbs, respectively. **h**, **i**, **j** Visualization of the different feature in four types of body-motion signals while the TENG is on the upper limbs, abdomen and lower limbs, respectively.


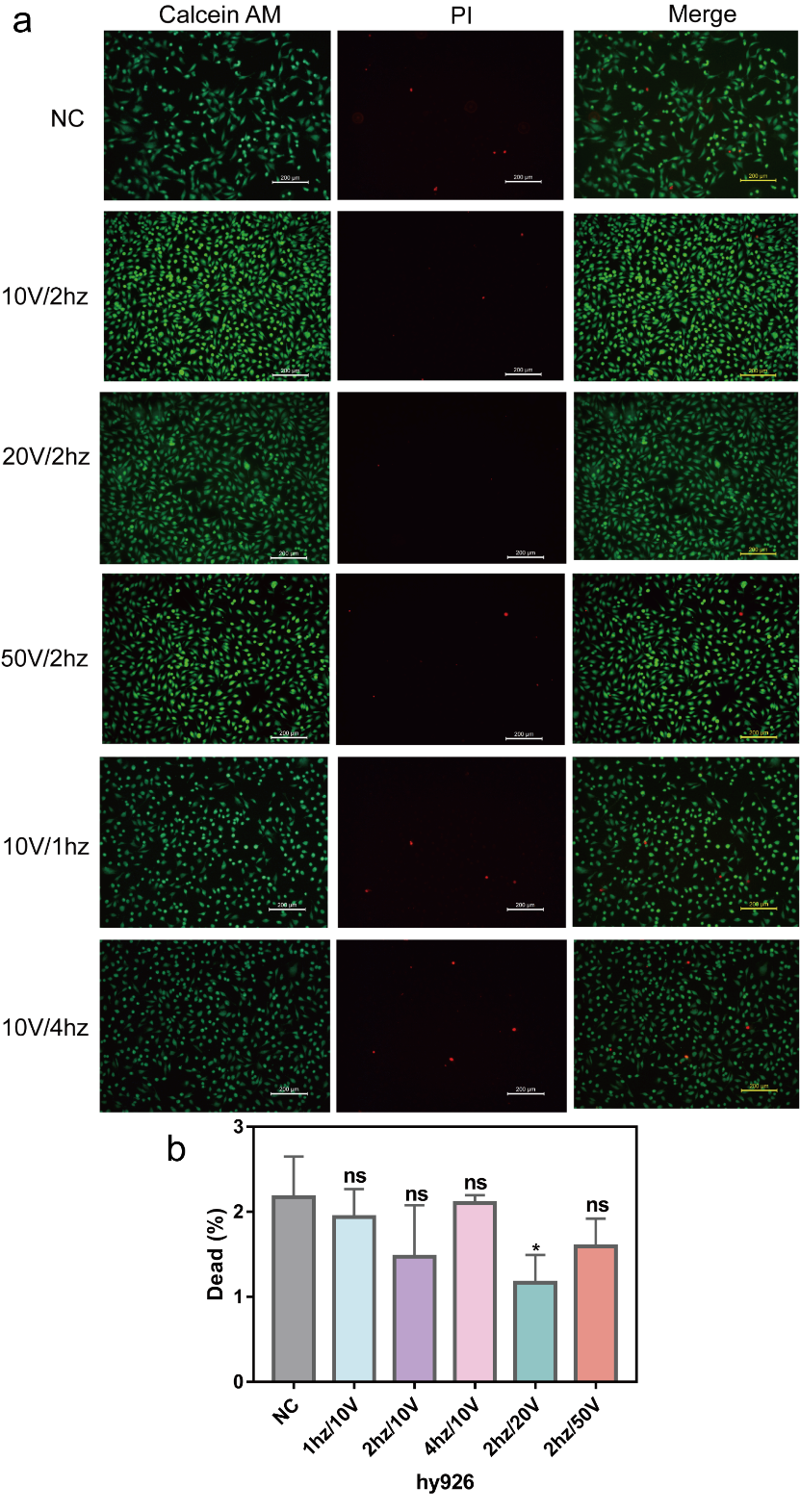


**Figure S7** Live/Dead Analysis for hy926 cells after TENG treatment. **a** Calcein AM/PI double staining fluorescence image of hy926 cells after treatment with different output voltage (10 V, 20 V, 50 V) and different frequencies (1 Hz, 2 Hz, 4 Hz); Magnification: ×200. **b** quantification result.


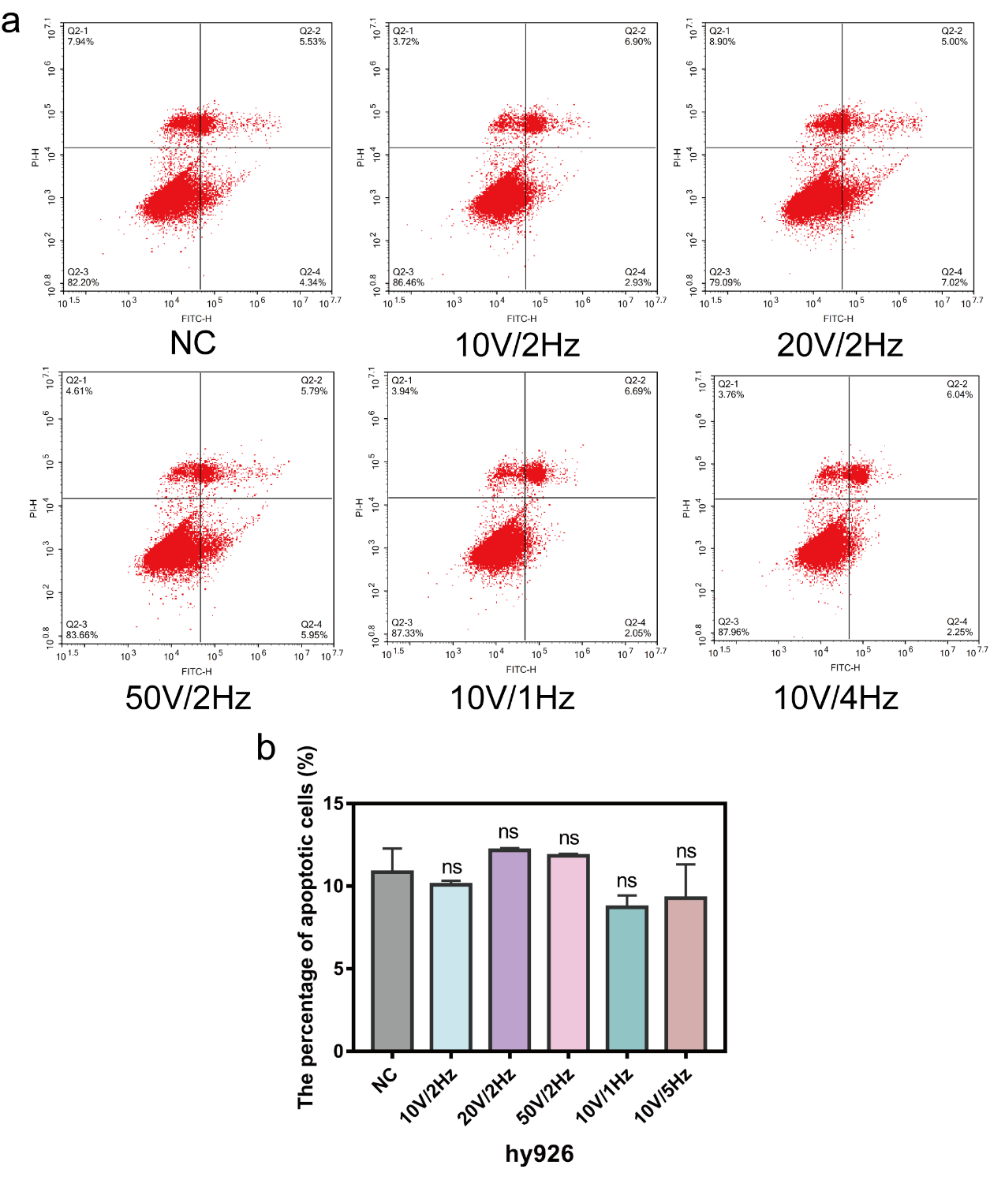


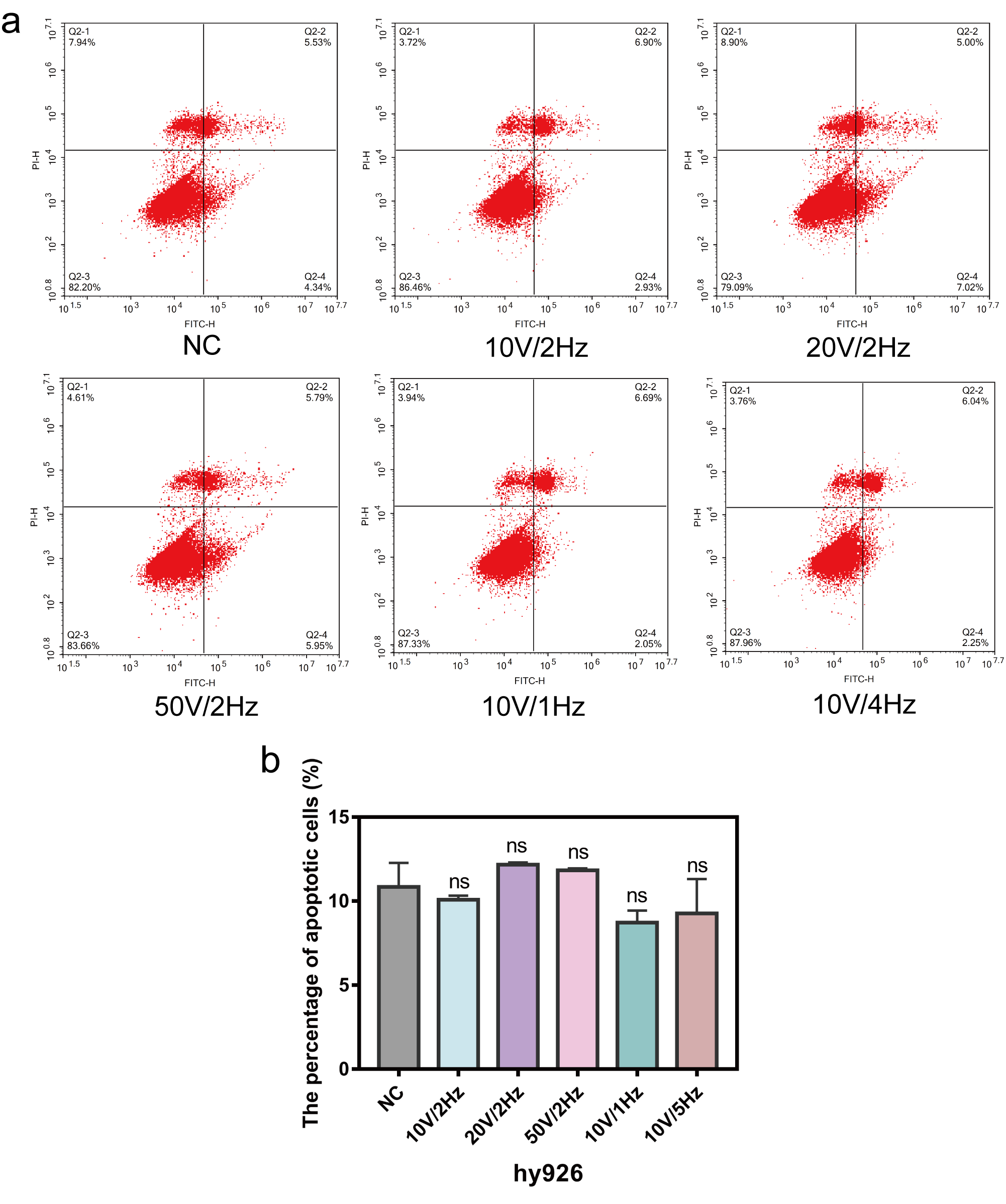


**Figure S8** Effect of different TENG output on apoptosis in hy926 cells. **a** Annexin V-fluorescein isothiocyanate (FITC) and propidium iodide (PI) staining was used to analyze the early and late apoptotic cell death in hy926 cells treated with different output voltage (10 V, 20 V, 50 V) and different frequencies (1 Hz, 2 Hz, 4 Hz). **b** Quantification result.


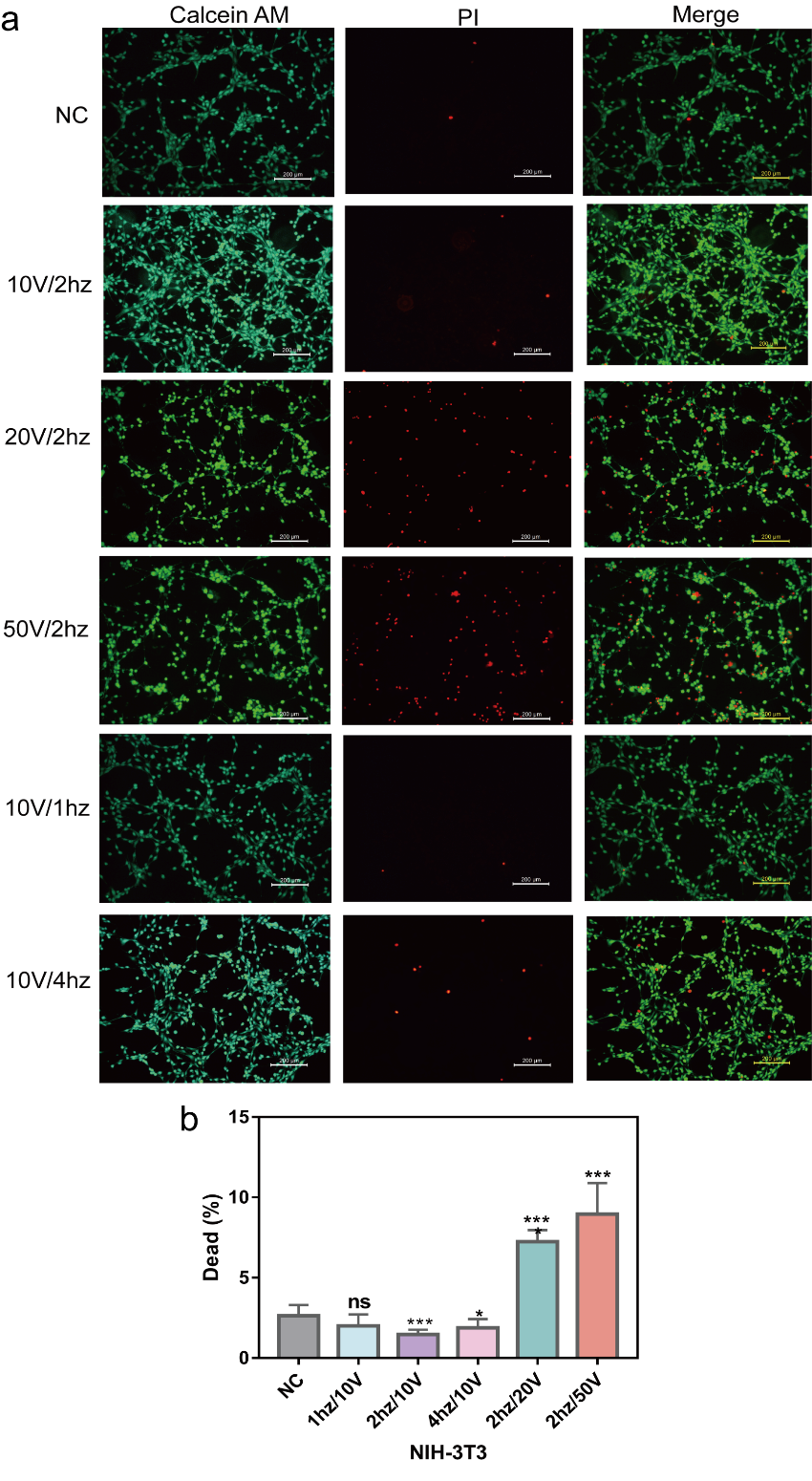


**Figure S9** Live/Dead Analysis for NIH-3T3 cells after TENG treatment. **a** Calcein AM/PI double staining fluorescence image of NIH-3T3 cells after treatment with different output voltage (10 V, 20 V, 50 V) and different frequencies (1 Hz, 2 Hz, 4 Hz); Magnification: ×200. **b** quantification result.


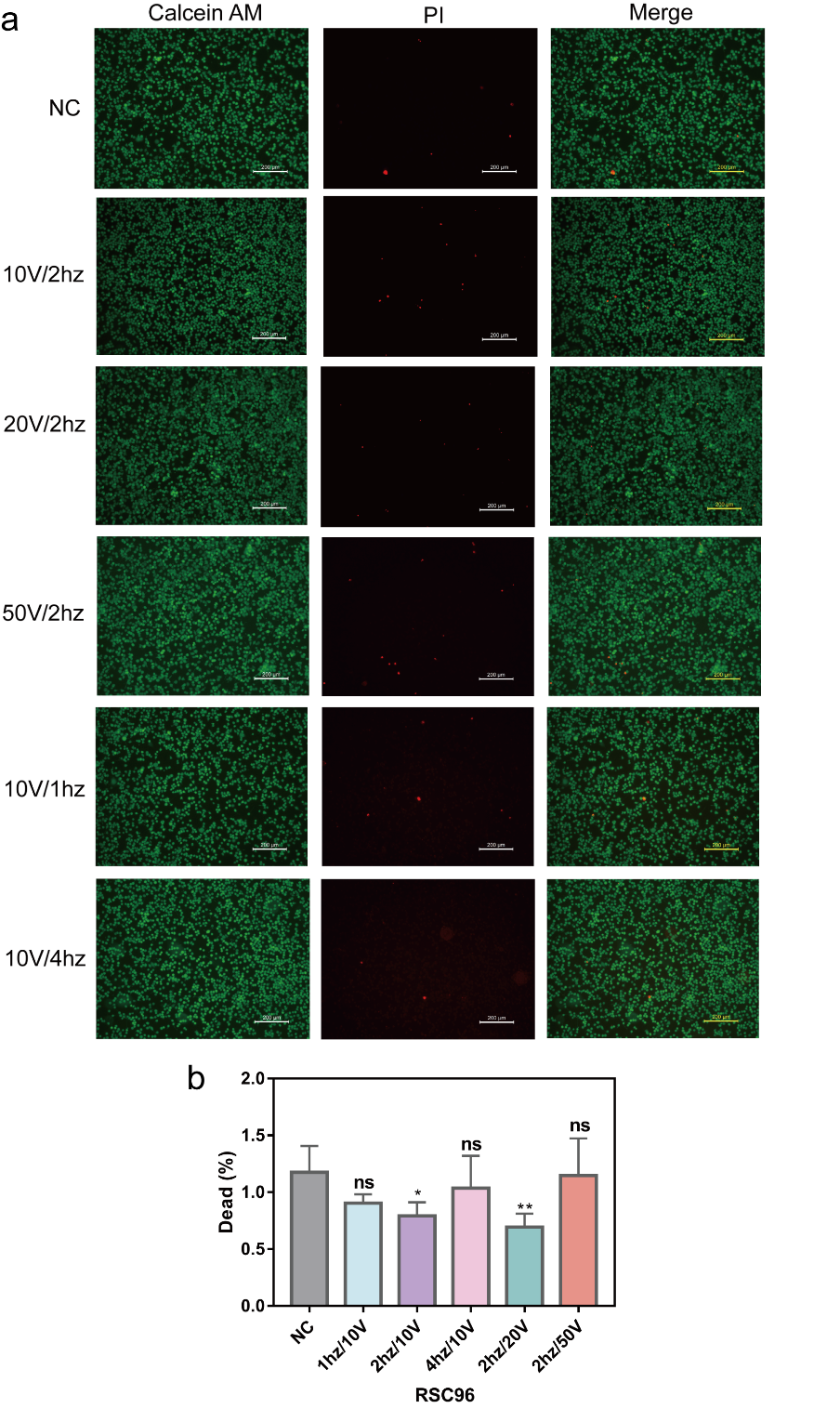


**Figure S10** Live/Dead Analysis for RSC96 cells after TENG treatment. **a** Calcein AM/PI double staining fluorescence image of RSC96 cells after treatment with different output voltage (10 V, 20 V, 50 V) and different frequencies (1 Hz, 2 Hz, 4 Hz); Magnification: ×200. **b** quantification result.


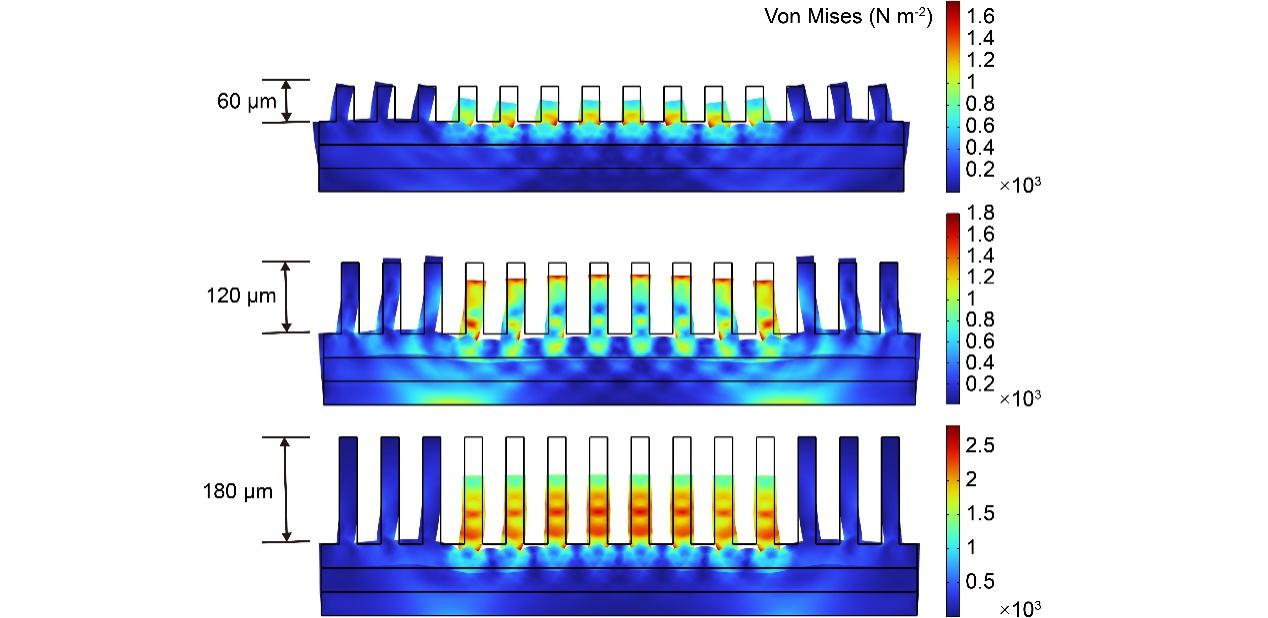


**Figure S11** Deformation simulation in COMSOL software with varying micropillar sizes. Utilizing actual scene parameters and material properties, the solid mechanics module of COMSOL was employed to simulate the Von Mises generated by micropillars with heights of 60, 120, and 180 µm under identical force application. The assessment of vertical force conversion efficiency was based on the force exerted by each micro-column. It can be seen from the simulation results that the 180 µm high micro-column exhibits a greater Von Mises value, indicating its ability to generate a more substantial vertical force. Consequently, this heightened force contributes to a more comprehensive contact and separation of the underlying film.
